# Supplementary figures and images for: A shotgun metagenomic investigation of the microbiota of udder cleft dermatitis in comparison to healthy skin in dairy cows
Source: PLoS One. 2020 Dec 2;15(12):e0242880. doi: 10.1371/journal.pone.0242880 (PMC7710049; doi:10.1371/journal.pone.0242880)

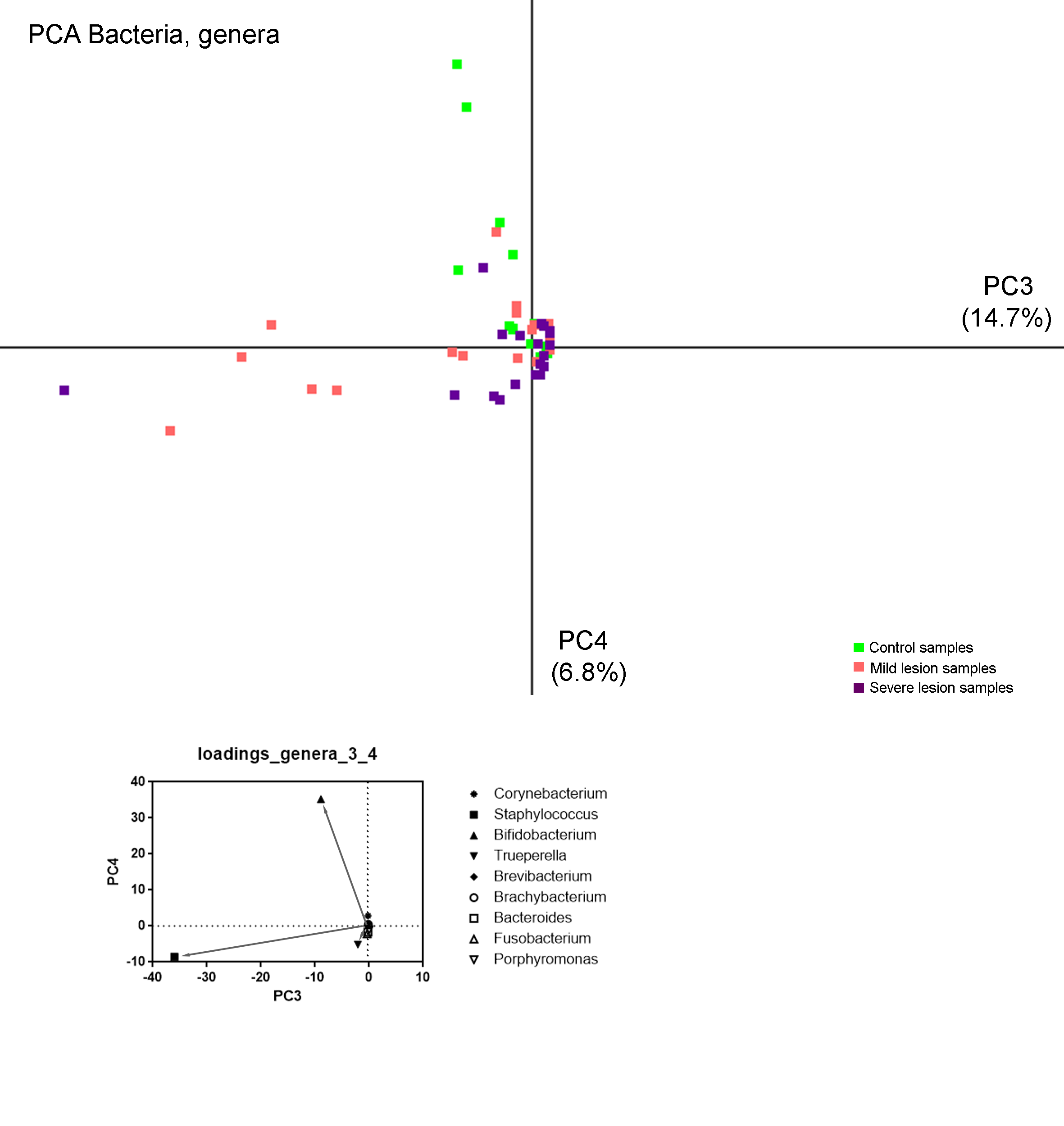

Supplement: S2 Fig — (TIF) [file pone.0242880.s004.tif]

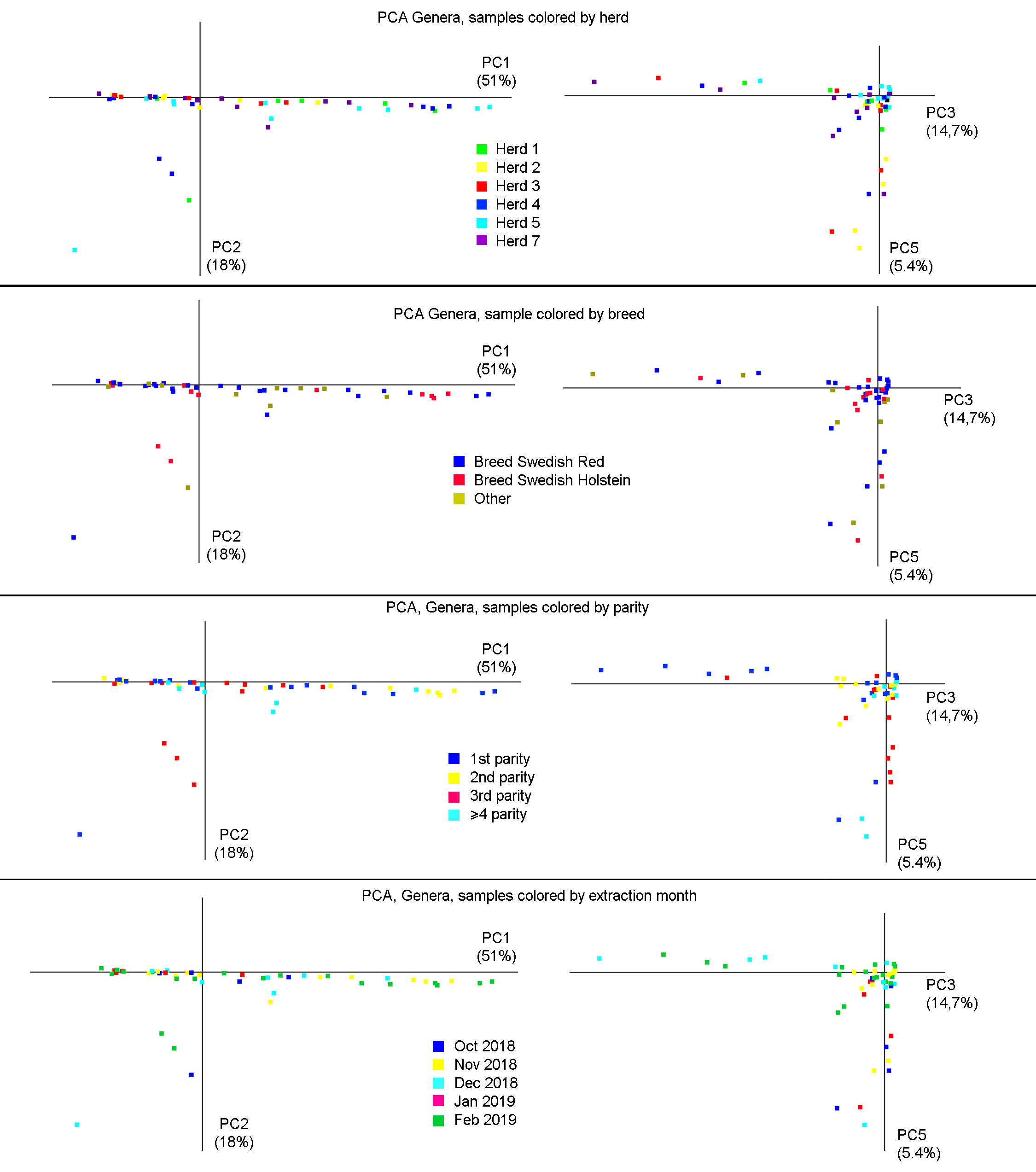

Supplement: S3 Fig — (TIF) [file pone.0242880.s005.tif]

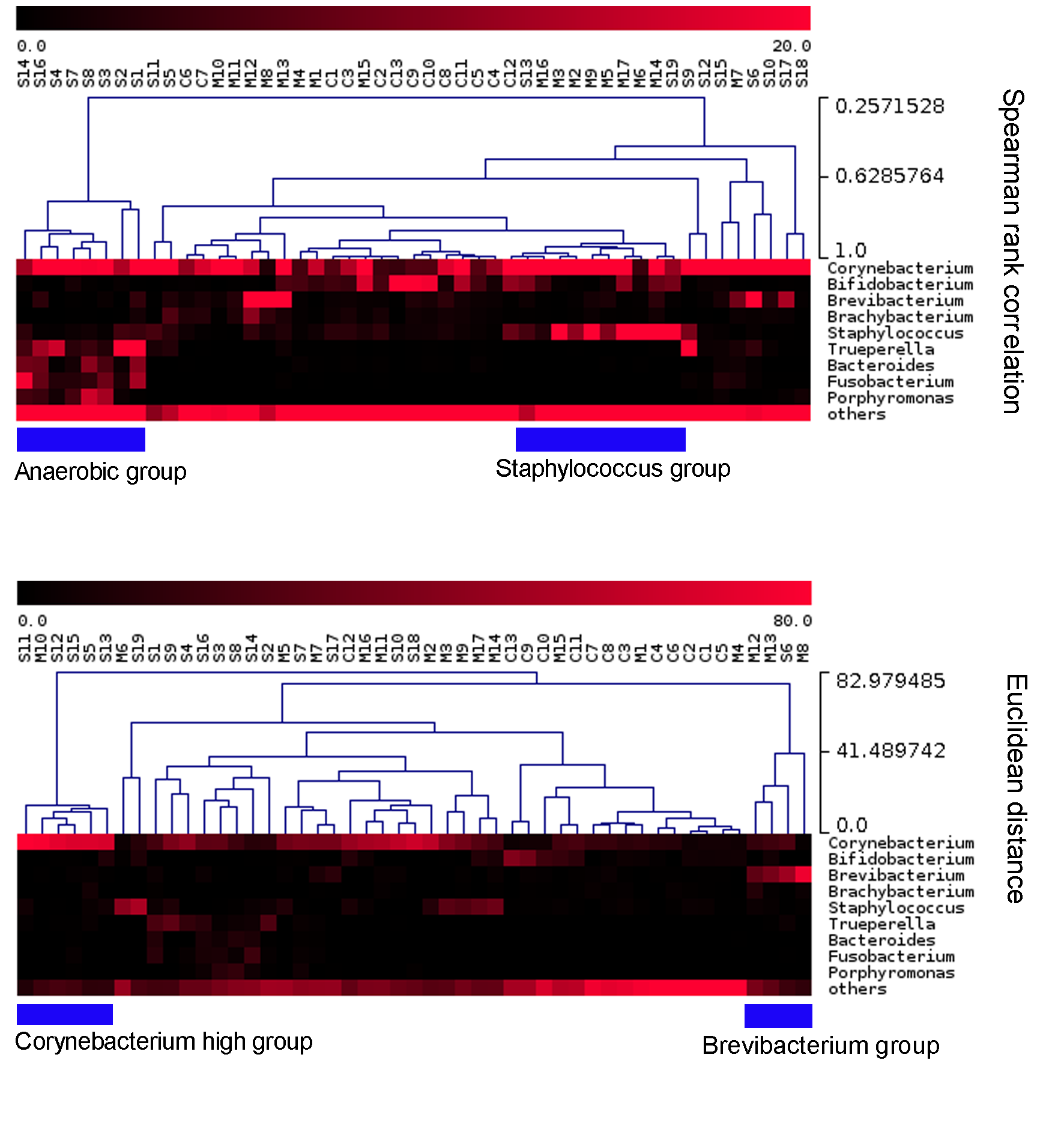

Supplement: S4 Fig — (TIF) [file pone.0242880.s006.tif]
