# Supplementary material for: A shotgun metagenomic investigation of the microbiota of udder cleft dermatitis in comparison to healthy skin in dairy cows
Source: PLoS One. 2020 Dec 2;15(12):e0242880. doi: 10.1371/journal.pone.0242880 (PMC7710049; doi:10.1371/journal.pone.0242880)
Supplement: Attachment — Submitted filename: Response_to_Reviewers.docx [file pone.0242880.s007.docx]

**Response to reviewers**

*We thank the reviewers for their relevant comments and help with improving the manuscript. As a result of the trimming of sequences, and to be able to perform the Bracken estimations, we re-analyzed our sequence reads using an updated Kraken2 database which led to some additional differences in the results that can be seen in the revised manuscript. We also decided to change the criteria for the bacteria presented in Table 1, corresponding to the criteria for bacteria presented in Fig 2 and 4, to make the manuscript more coherent.*

Reviewer #1: Ekman et al. investigate the skin microbiome of healthy and diseased cows using a shotgun metagenomic sequencing approach. Although I believe the study to have merit, it does contain a number of methodological flaws that make it difficult to evaluate in its current form.

Major

DNA extraction, library preparation and sequencing

Negative controls are an important component for any type of experiment where measurements are being made. The importance of these and their impact have been documented extensively throughout the microbiome literature (see [https://bmcbiol.biomedcentral.com/articles/10.1186/s12915-014-0087-z](https://eur01.safelinks.protection.outlook.com/?url=https%3A%2F%2Fbmcbiol.biomedcentral.com%2Farticles%2F10.1186%2Fs12915-014-0087-z&data=02%7C01%7Cbo.segerman%40sva.se%7C67cdc13d1913449787fd08d849890463%7Cb64756aa7df04f2ea41171214a50af24%7C0%7C0%7C637340200149016104&sdata=sXU60HFlPC3jkKVdoclW7k7GqUWReMqvgP0uFJif25A%3D&reserved=0)). Because they were not included in this experiment, it is an obvious limitation that should be discussed, but I also think that their impact depends on how you analyze and present results. For example, if you make claims about specific OTUs, then you should be confident that what you are describing is real, but if you make claims about general trends at a specific taxonomic level (e.g., phylum, genus, species, etc..), as you do here, then contamination may have less of an impact on the overall results, especially when DNA yield is high and contaminants are in low abundance. Please address the importance of negative controls in microbiome research and discuss the limitations of not including them in your study.

*We have added a short discussion on this limitation in the final remarks.*

It is also not clear how putative batch effects were handled in this study. Were all samples extracted on the same day? Were they sequenced on the same date, on the same machine?

*We have added information about extraction day in S1 Table. All sequencing was done in a single novaseq-run. To roughly determine that DNA extraction date did not interfer with the identified subgroups of samples we colored the samples by extraction month (which is also related to sampling date) in the PCA plot for the bacterial genus level and found no evidence of such interference (Fig S3).*

Bioinformatics

Overall, I think that the methods describing your bioinformatic methods is very well done; however, I do have a concern regarding the omission of how adapter sequences and low quality sequences were handled. Adapter contamination is known to affect alignment statistics for commonly used sequence aligners like BWA and Bowtie2 ([https://academic.oup.com/bioinformatics/article/30/15/2114/2390096](https://eur01.safelinks.protection.outlook.com/?url=https%3A%2F%2Facademic.oup.com%2Fbioinformatics%2Farticle%2F30%2F15%2F2114%2F2390096&data=02%7C01%7Cbo.segerman%40sva.se%7C67cdc13d1913449787fd08d849890463%7Cb64756aa7df04f2ea41171214a50af24%7C0%7C0%7C637340200149016104&sdata=Q5k88u44kQXcKhuxXhvRFE4IGbBoKDC7G0HUOhD9gF0%3D&reserved=0)), resulting in reads that are incorrectly classified as “unmapped”. This does appear to have affected the reported proportions of “host” versus “non-host” DNA for severe UCD samples and others, as a large proportion of your sequence reads were classified to the Babesia bigemina genome, which I know to suffer from Bos taurus DNA contamination. Failure to remove these adapter sequences may have also affected classification statistics for other sequences processed with Kraken2. Please rerun your raw sequence data through a read trimming tool like Trimmomatic, cutadapt or other relevant tool before proceeding with the remainder of your analysis. Please also add a column to Supplementary Table 1 describing the number of sequence reads that survived this trimming and other quality control steps.

*Indeed, there was a fraction of the reads that had adaptor content. We have run all fastq files through trimmomatic to remove them. There were very few reads dropped during this procedure, but up to ~15% of the reads were shortened by the trimming. The kraken analysis was then updated. We used a new fresh build of the kraken database in this step and added a Bracken analysis. We have updated the bioinformatic description in the method section and the information in table S1.*

Statistics

Breed, herd, and parity are likely confounders that should be included in your statistical analysis. Please consider accounting for these potential confounders and update your methods.

*We agree that especially herd, but also breed and parity, may have an effect on the microbiota. To investigate the distribution of these factors over the control and UCD groups we performed Fisher’s exact test and found that these factors did not differ significantly between the groups (this information has been updated in the manuscript). As we wanted to compare the bacterial abundance between groups and as the number of observations were limited (and the bacteria far from normally distributed within samples), we believe that a multivariable analysis is less useful for this dataset. As a spot check, we did test a mixed effect ordinal logistic regression model with bacterial abundance as an ordinal outcome for analyzing some of the most abundant species and found that the estimates (and significance levels) for UCD status was largely unaffected when these potential confounders were included (although herd was significantly associated with e.g. Bifidobacterium abundance). Thus, we would like to keep the Wilcoxon rank sum tests in the manuscript.*

*In addition, to visually interpret if any of the above mentioned variables had any substantial impact on the clustering of samples in the PCA, we colored the samples by herd, breed and parity in the PCA plot for the bacterial genus level, and found no evidence of such associations (FigS3).*

Minor

Title: Please consider changing the title, as this seems to be a study comparing the microbiome of healthy and diseased skin (i.e., UCD).

*We have changed the title.*

Line 66: Sequencing methods (e.g., 16S amplicon sequencing, shotgun sequencing, etc..) are all biased in some way, as are the sequence databases used to describe the underlying results. You do an excellent job of discussing the tradeoffs of each approach later in the manuscript, but I recommend removing this sentence as it is inaccurate.

*We have removed this sentence.*

Line 77: Shotgun metagenomics can also reveal strain-level information. Please add this and a relevant citation.

*We have added this and included a recent review on this topic to the citations.*

Line 102: Change “All scoring and sampling were performed by the first author” to “All scoring and sampling was performed by a single researcher”.

*Changed according to suggestion.*

Line 119: Was a pre-dipping solution applied prior to milking? If so, please add and specify relevant product information.

*The majority of herds did not use any pre-dipping solution, although all of them used some kind of teat spray or dip post-milking. We do not have any information on the products that were used, but since the UCD lesions are located rather far from the teats, and as all cows within a herd (controls and UCD cows) received the exact same products and procedures during milking, we do not believe that this had any effect on the results of the study.*

Line 215: Please clarify what you mean by “complexity”. What does it mean for the complexity of a sample to be estimated and why was this done?

*We agree that this analysis is somewhat difficult to interpret. We have removed this from the manuscript (including Fig 5C).*

Line 220: Please list the bacterial species that were included in this analysis.

*We have indicated these bacterial species in the S2 Table (Bracken results presented in percentages).*

Line 248: Please clarify what is meant by “... there was no convincing trend …” Was this based on a pre-determined p-value or “eyeball method”?

*We have updated the information of fungal reads in the manuscript. There were no significant differences between sample types in the proportion of classified reads for protozoa and viruses, we have now added this information in Figure 2. In addition, a brief “eyeball method” of the table of classified viral and protozoan reads gave no indication that any taxa were more abundant in any specific sample type.*

Line 270: How are the samples labeled on the ordination plots? It seems that some of the points/samples have labels, but others do not. What is the criterion for labeling samples?

*To make a more unbiased presentation of the data, all samples have now been indicated in the plots.*

Lines 476-477: The design of this study was probably not sufficient to determine “causation” of dysbiosis; consider re-wording this sentence and others (e.g., 567-569).

*We have changed the wording in this sentence to clarify. In our understanding, a decreased diversity is indicative of an unbalanced microbiota, and thus a dysbiosis, so it was not our intention to assume any causation. We also added a sentence in the Conclusions section to clarify this.*

Line 555: Delete “Unbiased”. I know what you mean here, but there are numerous other sources of bias in microbiome studies. The choice of sampling device, the choice of DNA extraction kit, the choice of DNA shearing method (e.g., enzymatic versus mechanical), the choice of sequencing depth (e.g., shallow versus deep), and the choice of reference sequence database can all bias shotgun metagenomic experiments.

*We have deleted the word unbiased.*

**Reviewer #2:** The primary goal of this manuscript is to compare the cutaneous microbiome of sites with mild/severe dermatitis lesions to healthy skin of the fore udder attachment. To accomplish this, the authors generated shotgun metagenomic sequencing data for 49 samples. Analysis of these samples with the k-mer approach Kraken revealed differential communities between controls and samples with UCD. Notably, subgroups existed within the UCD samples and few known mastitis-causing pathogens were identified. Refreshingly, they looked across kingdoms and not only at bacteria. While authors have generated a valuable dataset and the overall methodology is technically sound, there are a few areas that could be improved.

Methods:

- The authors utilized Kraken to classify their metagenomic sequencing reads and then directly used these results to draw conclusions about which genera and species were differential between groups. Because Kraken classifies individual reads to their best matching location in the taxonomic tree and does not actually estimate the abundance of species, the program Bracken ([https://ccb.jhu.edu/software/bracken/](https://eur01.safelinks.protection.outlook.com/?url=https%3A%2F%2Fccb.jhu.edu%2Fsoftware%2Fbracken%2F&data=02%7C01%7Cbo.segerman%40sva.se%7C67cdc13d1913449787fd08d849890463%7Cb64756aa7df04f2ea41171214a50af24%7C0%7C0%7C637340200149026102&sdata=7wFkKvdRsj5A5Ig0hxWh1nQDtTat3BpFg1x%2FkXUxK%2BY%3D&reserved=0)) should be applied to the Kraken results and then the Bracken species abundances should be compared between groups.

*We have run the data through Bracken to estimate species, genera and phylum level data. We have updated the manuscript to use Bracken data instead of Kraken data. We had to build a new Kraken database and reanalyze the data, since the previously used database lacked files required by Bracken.*

Figure 3:
- Are any of the UCD subgroups, driven by the herd of the animal?

*We agree that herd (and also breed and parity), may have an effect on the microbiota. To visually interpret if any of the these variables had any substantial impact on the clustering of samples in the PCA, we colored the samples by herd, breed and parity in the PCA plot for the bacterial genus level, and found no evidence of such associations (FigS3).*

*In addition, to investigate the distribution of herd, breed and parity over the control and UCD groups we performed Fisher’s exact test and found that these factors did not differ significantly between the groups (this information has been updated in the manuscript). As we wanted to compare the bacterial abundance between groups and as the number of observations were limited (and the bacteria far from normally distributed within samples), we believe that a multivariable analysis is less useful for this dataset. As a spot check, we did test a mixed effect ordinal logistic regression model with bacterial abundance as an ordinal outcome for analyzing some of the most abundant species, with herd as random factor, and found that the estimates (and significance levels) for UCD status was largely unaffected when these potential confounders were included (although herd was significantly associated with e.g. Bifidobacterium abundance).*

- In the "Bacterial abundance" section, the authors discuss several of the taxa that are driving these ordinations. Can the authors add biplot lines for these driver taxa to the PCA plots?

*In our opinion the figure got too dense with biplot lines, but we have added a supplemental figure with loadings for the PC axis used (S2 Fig).*

-For the species based results (Fig 3C), are similar conclusions drawn if the authors perform ordination analysis on a distance matrix, calculated with Bray Curtis, on the samples? It's unclear how there is so little variance at the species level between the control samples.

*The control samples did not show a large variability for the taxa which were the main drivers for the displayed PC axes. However, the control samples do spread out in projections with higher PCs. We do not have the Bray Curtis distance option in the PCA tool used. We have tried to plot a Bray Curtis based ordination in R. The control samples cluster in there as well, although not fully as tight as in the PCA, and UCD samples cluster in roughly similar patterns as in Fig 3C.*

Figure 4:
- The authors frequently describe different UCD subgroups. To complement these descriptions, can the authors utilize a hierarchical clustering based method to more robustly define them?

*We have added a hierarchical clustering analysis to the supplement (S4 Fig).*

Minor comments:

- In Fig 2A, the red/green coloring of Archaea and Fungi will be difficult for colorblind individuals to distinguish. Can the authors please update?

*We have changed the colors.*

- To make Fig 2B more informative, can the authors use individual scales for the different kingdoms and denote p-values for those comparisons that are significant?

*We have made an enlargement for the minor taxa and added the p-values.*
